# Acupuncture for Cancer-Induced Bone Pain in Animal Models: A Systemic Review and Meta-Analysis

**DOI:** 10.1155/2020/5606823

**Published:** 2020-07-30

**Authors:** Mei-ling Yu, Jia-jia Qian, Shu-ping Fu, Jia-ying Chen, Yu-wen Zheng, Zhi-gang Lu, Sheng-feng Lu

**Affiliations:** ^1^First Clinical Medical College, Nanjing University of Chinese Medicine, Nanjing 210023, China; ^2^Acupuncture and Rehabilitation College, Nanjing University of Chinese Medicine, Nanjing 210023, China; ^3^Key Laboratory of Acupuncture and Medicine Research of Ministry of Education, Nanjing University of Chinese Medicine, Nanjing 210023, China; ^4^College of Pharmacy, Nanjing University of Chinese Medicine, Nanjing 210023, China

## Abstract

**Background:**

Cancer-induced bone pain (CIBP) is a highly prevalent symptom, which afflicts vast majority of patients who suffer from cancer. The current treatment options failed to achieve satisfactory effect and the side effects were prominent. Recent randomized controlled trials (RCTs) of animal demonstrate the benefit of acupuncture for CIBP. We sought to determine if the pooled data from available RCTs supports the use of acupuncture for CIBP.

**Methods:**

A literature search for randomized controlled trials was conducted in six electronic databases from inception to May 31, 2019. Meta-analysis was performed with Review Manager 5.3 software; the publication bias was assessed by Stata 12.0 software. We used random effects model for pooling data because heterogeneity is absolute among studies to some extent.

**Results:**

Twenty-four trials were included in the review, of which 12 trials provided detailed data for meta-analyses. Preliminary evidence indicates that compared to wait list/sham group, acupuncture was effective on increasing paw withdrawal threshold (PWT) and paw withdrawal latency (PWL). Compared to medicine, acupuncture was less effective on PWT, but as effective as medicine on PWL. Acupuncture can reinforce medicine's effect on PWT and PWL. Compared to the control group, acupuncture was superior to increase body weight (BW), decrease spinal cord glial fibrillary acidic protein (GFAP), and interleukin-1*β* (IL-1*β*). Furthermore, some studies showed acupuncture delay or partially reverse morphine tolerance. Three studies found acupuncture has no effect on PWT, but 2 of them found acupuncture could enhance small dose of Celebrex's effect on CIBP.

**Conclusions:**

Acupuncture was superior to wait list/sham acupuncture on increasing PWT and has no less effect on increasing PWL compared to medicine; acupuncture improved the efficacy of drugs, increased the CIBP animals' body weight, and decreased their spinal cord GFAP and IL-1*β*. High-quality studies are necessary to confirm the results.

## 1. Introduction

Cancer-induced bone pain (CIBP) refers to bone pain caused by primary bone neoplasm or secondary to other carcinomas. It is a highly prevalent symptom, which afflicts vast majority of patients who suffer from cancer [1]. Primary bone cancers just account for less than 1% of diagnosed cancers every year [2]; bone metastasis, which greatly increases patient's risk of fractures and other structural complications, is the most common contributor of cancer-related pain [3]. Bone pain limits patient's mobility; increases depression and anxiety; and increases risk of lung infection, cutaneous ulcers, and vein thrombosis [4]. Effective treatment of CIBP is urgent as the patients' survival increases due to rapid development of new available target treatments.

Up to now, the three-step ladder World Health Organization (WHO) model is still the most frequently used program for cancer pain clinically. Opioids remain considered as one of the most effective pain-relieving therapeutics. Oral morphine is the first choice in patients who can assume oral drugs [5]. However, the long-term use of opioids is limited by the development of tolerance, potential for addiction and hyperalgesia [6–8]. Besides, up to 40% of cancer patients with no previous emesis complained of opioid-induced nausea and vomiting; meanwhile, opioid-related constipation and center nervous system (CNS) symptoms deserve attention [5]. It should be notice that, compared with other pain states, the analgesic effects of opioids can be less effective in CIBP [9–11]. Evidence indicates that at least nearly 25.5%–31% of patients have been troubled by severe pain and do not get satisfactory treatment, while undertreatment of cancer pain was negatively associated with patients' overall well-being [12–14]. Furthermore, about 70% of patients reported side events associated with their pain medication, which impose negative impact on activities of daily living [13]. Based on the above reasons, patients and clinicians aspire to better treatment with definite effects and fewer side effects.

As one of the traditional Chinese medicines, acupuncture is known for its effect on pain-relieving in cancer therapy and almost free of adverse effect. Plenty of studies have proven that acupuncture is effective on some cancer-induced pain, such as pain arising from hepatocellular carcinoma [15], stomach carcinoma [16], ovarian cancer [17], pancreatic cancer [18], breast cancer [19], and so on. Can acupuncture be used to alleviate CIBP? Clinical study in this area is rare, but it is worth to notice that animal research is gradually increasing. To clarify the potential benefits of acupuncture for CIBP in animals, we conducted this systematic review and meta-analysis. The review protocol was registered with PROSPERO (CRD42019133175).

## 2. Material and Methods

### 2.1. Inclusion and Exclusion Criteria

We carried out the systematic review following Cochrane and preferred reporting items for systematic reviews and meta-analysis (PRISMA) guidelines. The studies were included if they explored acupuncture's effect on cancer-induced bone pain. Stimulation on acupoint, whether using needle insertion or other mediators, was accepted. Different types of acupuncture comparison were excluded. Randomized controlled trials were eligible for inclusion. Studies were included in the analysis more than once if they had different arms. If more than one scheme was employed, only the most effective was used. The primary measures of treatment benefit were PWT and PWL. BW (body weight), spinal cord glial fibrillary acidic protein (GFAP), and interleukin-*β* (IL-*β*) were chosen as the secondary outcomes. Studies that did not include the primary or secondary outcomes were excluded. The primary exclusion criteria were observational nonrandomized studies, ongoing trials without results, case reports, reviews, and duplicate studies. Studies that did not include a usual-care and/or placebo comparison group and for which a final data analysis was not available were excluded.

### 2.2. Search Strategy

Briefly, we searched PubMed, Embase, China National Knowledge Infrastructure (CNKI), Chinese Science and Technology Periodical Database (VIP), Wanfang database, and China Biomedical Literature Service System (SinoMed). Literature searches were conducted from their inception up to May 31, 2019. MeSH terms for PubMed and comparable terms for other databases were employed during search. The following search terms were included in various combinations: “manual acupuncture”/“electro-acupuncture(EA)”/“five-element acupuncture”/“auricular acupuncture”/“auricular needle”/“laser needle”/“transcutaneous acupoint electrical stimulation”/“catgut implantation”, and “cancer-induced bone pain”/“bone tumor”/“bone marrow cancer”/“multiple myeloma”/“bone neoplasm”/“osseous metastasis”/“bone metastasis”/“skeletal metastases”/“bone cancer”. All timings, frequencies, and durations of treatment are eligible for inclusion. Bibliographies of relevant papers were screened. Searches were limited to animal studies published in English and Chinese. Case reports, editorials, letters, comments, or any types of reviews were excluded.

### 2.3. Data Screening and Extraction

All studies that met the inclusion criteria were arranged to data screen and extract. Two reviewers (Mei-ling Yu and Jia-jia Qian) screened titles and abstracts for initial inclusion in an independent manner, and disagreements were resolved by discussion and reviewing the full paper. Two reviewers (Jia-ying Chen and Yu-wen Zheng) performed data extraction on the included trials, and disagreements were settled by consulting a third reviewer (Mei-ling Yu).

### 2.4. Evaluation of Risk of Bias

Study quality was evaluated with Systematic Review Centre for Laboratory Animal Experimentation (SYRCLE) and Standard for Reporting Interventions in Clinical Trials of Acupuncture (STRICTA) by independent investigators (Sheng-feng Lu and Jia-ying Chen). We used the SYRCLE's risk of bias (RoB) tool to assess risk of bias of the included literature. The following contents are contained [20]: (1) selection bias; (2) performance bias; (3) detection bias; (4) attrition bias; (5) reporting bias; and (6) other sources of bias. The STRICTA checklist was employed to assess the quality of acupuncture trials. The following contents are contained [21]: (1) acupuncture rationale; (2) details of needling; (3) treatment regimen; (4) other components of treatment; (5) practitioner background; and (6) control or comparator interventions.

### 2.5. Statistical Analysis

Given that heterogeneity is inevitable across studies, random effects meta-analysis was used to synthesize results. *I*^2^ was employed to quantify the heterogeneity (*I*^2^<25%—low, 25–50%—moderate, and >50%—high degree of heterogeneity). We conducted sensitivity analyses to explore the robustness of our results and to evaluate whether any of the included studies had a large influence on the results. Data were summarized by standard mean difference (SMD) with 95% CI for continuous outcomes. A *χ*^2^-based test of homogeneity was performed and the inconsistency index (*I*^2^) and *Q* statistics were determined. Pooled effects were calculated and a 2-sided *P* value <0.05 was considered to indicate statistical significance. The leave-one-out approach was used for sensitivity analysis of the primary outcome. Publication bias was assessed by funnel plot and egger's test. Analysis was conducted in Review Manager 5.3 and Stata 12.0 software.

## 3. Results

### 3.1. Study Selection

**Figure 1:**
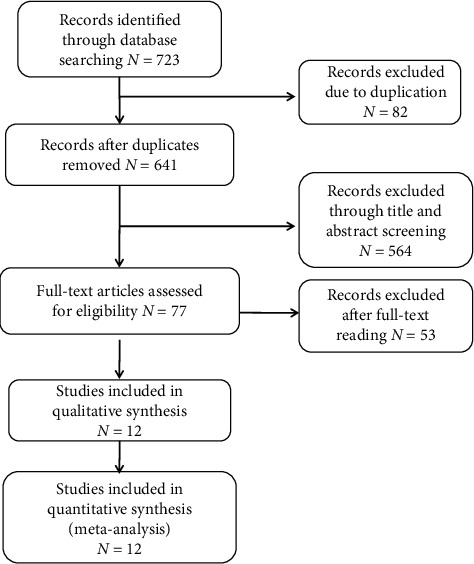
Flow diagram for study selection.

A total of 723 articles were identified with the search terms. 82 duplicates were omitted, and the left 641 publications were screened. 564 studies were excluded for at least one of the following reasons after title and abstract were screened: (1) the intervention did not include acupuncture; (2) the stimulation site was not at the acupoint; (3) the model was not cancer-induced bone pain; (4) they were not animal studies; (5) they were reviews or case reports or letters; (6) the outcomes did not include PWT/PWL/BW/GFAP/IL-1*β*; and (7) they did not include a usual-care and/or placebo comparison group. 77 articles remained after initial reading. Then, we read the full papers: 53 papers were excluded and 24 articles met the inclusion criteria finally (Figure 1). Among them, 3 papers were from Ph.D. dissertations. By reading the full texts of the included studies, we found some studies investigating acupuncture effects from various aspects, so they were divided into more than one RCT according to their arms. Finally, the meta-analysis was divided into 3 parts: (1) acupuncture with control; (2) acupuncture with western medicine; and (3) combination of acupuncture and medication with medication.

### 3.2. Study Characteristics

The detailed information of the animal model is provided in Table 1. 977 animals were involved totally. 12 studies used female SD rats; 3 studies used male SD rats; 6 studies used female Wistar rats; male Copenhagen rat was employed in 2 studies; and BALB/c mice were used in 1 study. 19 studies used walker256 mammary gland carcinoma cells to made model; 2 studies used MRMT-1 mammary gland carcinoma cells; 2 studies used AT 3.1 prostate cancer cell line; and 1 study used K7M2 osteosarcoma cell strain.

The acupuncture treatment parameters of the included studies are listed in Table 2. Among all the studies, a total of 8 points involving 4 channels, as well as the extra points, were used. The frequencies of acupuncture points from high to low were as follows: ST36, 19 times; BL60, 12 times; GB30, 4 times; L3-L5 EX-B2, 3 times; BL2, 2 times; SP6, 2 times; and GenDuan, 2 times. Needle retaining was for 30 min in 22 studies, 6.5 min in 1 study, and 13 min in 1 study. All studies used electroacupuncture (EA). The electrical frequency employed was 2/100 Hz in 15 studies; the rest frequency was decrement in turn: 15 Hz, 4 Hz, 10 Hz, 2 Hz, and 4/60 Hz. One study did not report the EA frequency. EA intensity of all the included studies was between 0.3 and 2.0 mA except for Li2010 [24]. The frequency of EA treatment was as follows: 15 studies provided acupuncture once every day; 7 studies provided acupuncture every other day; 1 study provided acupuncture 2 times per week; and 1 study was investigate the effect of continuum and interval treatment. The number of treatments ranged from 5 to 20 times, mainly focusing on 6 and 7 times. No serious adverse events have been reported.

### 3.3. Quality Assessment

Standard for Reporting Interventions in Clinical Trials of Acupuncture (STRICTA) [44] checklist was used to assess the study quality from the aspect of acupuncture intervention (Table 3). Of the 24 included studies, all studies provided detailed style of acupuncture, 23 provided the detailed reasoning for treatment, and all studies described the extent to which treatment was varied. 22 studies provide the number of needle insertions per subject per session and the names of points used. 14 studies described the depth of insertion. Seven studies described the response sought. All the included studies used electrical stimulation during needle retention. All the studies described the needle retention time. Just 1 study described the needle type, 11 studies did not provide detailed information, and the remaining 12 studies did not provide any information of the needle type. All the included studies described the number of treatment sessions, frequency, and duration of treatment sessions. All the included studies described the details of other interventions administered to the acupuncture group. None of the included studies described the setting and context of treatment, participating acupuncturists, rationale for the control, or comparator in the context of the research question, with sources that justify this choice. All the included studies provided precise description of the control or comparator.

Systematic Review Centre for Laboratory Animal Experimentation (SYRCLE) [45] risk of bias tool was applied to assess the methodological quality of the animal experiments (Table 4). We found that 6 studies describe detailed sequence generation, 15 studies just mentioned randomness but did not provide specific random methods, and 3 studies did even not mention randomization. All the include studies provided baseline characteristics. No included studies mentioned allocation concealment. 21 studies mentioned random housing of the animals. None of the included studies described caregivers and/or investigators blinded from knowledge of which intervention each animal received during the experiment. None of the studies assess outcome randomly. Four studies described that the outcome assessor was blinded. Eight studies were free of incomplete outcome data and 10 studies were influenced by incomplete outcome data mainly due to model failure; the pity is that they did not inform if the missing outcome data were imputed by appropriate methods. All the included studies were free of selective outcome reporting. Five studies were free of other sources of bias, 13 studies were unclear about their influence by other sources of bias, and 6 studies were influenced by other sources of bias significantly.

### 3.4. Results of Meta-Analysis

#### 3.4.1. Acupuncture's Effect on PWT

**Figure 2:**
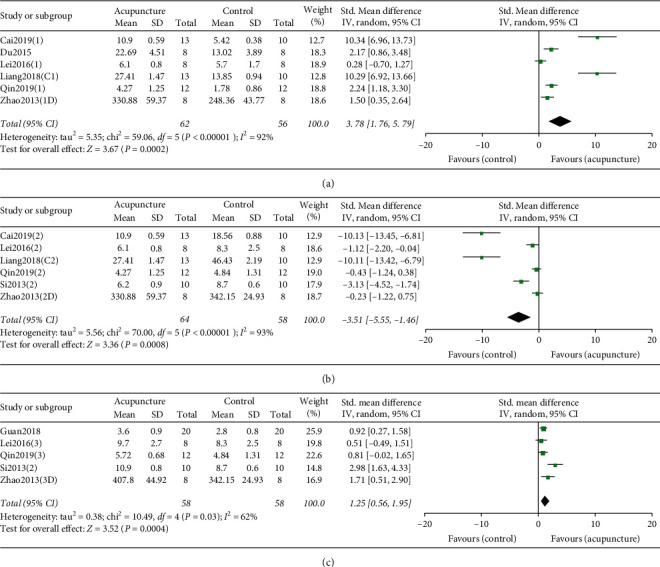
(a). Forest plot comparing paw withdrawal threshold between acupuncture and model. (b). Forest plot comparing paw withdrawal threshold between acupuncture and medicine. (c). Forest plot comparing paw withdrawal threshold between single medicine and medicine combined with acupuncture. The black diamond represents the point estimate and 95% confidence interval. The green block at the point estimate of intervention effect with a horizontal line extending either side of the block represents a study. The area of the block indicates the weight assigned to that study in the meta-analysis while the horizontal black line depicts the 95% confidence interval. Statistical method is inverse variance. Random effects model was employed as analysis model. The effect measure was standard mean difference.

*(1) Acupuncture versus Sham/Placebo Acupuncture or Wait List Group*. For a total of six RCTs based on the PWT to evaluate effect (Figure 2(a)), the pooled results showed that EA was prior to sham/placebo or wait list group, with mean difference of 1.50 (95% CI, 0.35 to 2.64; *P* < 0.00001; *I*^2^ = 92%).

*(2) Acupuncture versus Medicine*. Meta-analysis of six RCTs suggested PWT was significantly improved in control groups versus acupuncture groups (Figure 2(b)), with a mean difference of −3.51 (95% CI, −5.55 to −1.46; *P* < 0.00001; *I*^2^ = 93%).

*(3) Acupuncture Combined with Medicine versus Single Medicine*. Meta-analysis of five RCTs suggested a significant improvement in PWT in acupuncture combined with medicine groups versus single medicine group (Figure 2(c)), with a mean difference of 1.25 (95% CI, 0.56 to 1.95; *P* = 0.03; *I*^2^ = 62%).

#### 3.4.2. Acupuncture's Effect on PWL

**Figure 3:**
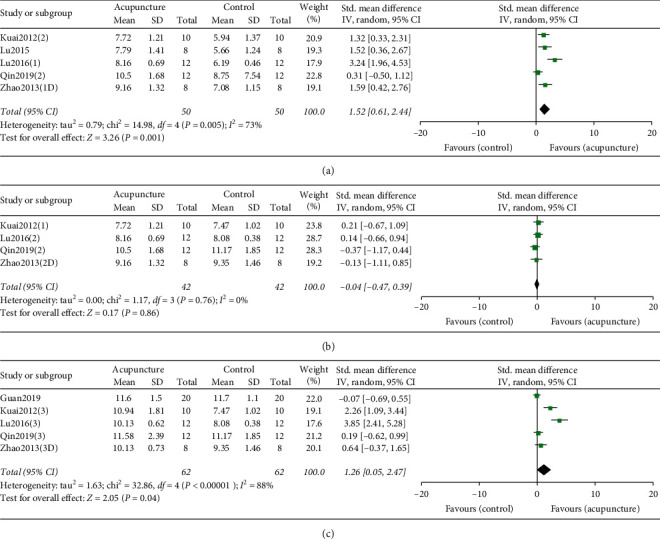
(a). Forest plot comparing paw withdrawal latency between acupuncture and model. (b). Forest plot comparing paw withdrawal latency between acupuncture and medicine. (c). Forest plot comparing paw withdrawal latency between single medicine and medicine combined with acupuncture. The black diamond represents the point estimate and 95% confidence interval. The green block at the point estimate of intervention effect with a horizontal line extending either side of the block represents a study. The area of the block indicates the weight assigned to that study in the meta-analysis while the horizontal black line depicts the 95% confidence interval. Statistical method is inverse variance. Random effects model was employed as analysis model. The effect measure was standard mean difference.

*(1) Acupuncture versus Sham/Placebo Acupuncture or Model*. Meta-analysis of five RCTs suggested PWL was improved significantly by acupuncture compared with sham/placebo acupuncture or wait list group (Figure 3(a)), with a mean difference of 1.52 (95%, 0.61 to 2.44; *P* = 0.005; *I*^2^ = 73%).

*(2) Acupuncture versus Medicine*. Meta-analysis of four RCTs showed that acupuncture had no less effect than medicine in terms of improving PWL (Figure 3(b)), with a mean difference of −0.04 (95%, −0.47 to 0.39; *P* = 0.76; *I*^2^ = 0%).

*(3) Acupuncture Combined with Medicine versus Single Medicine*. Meta-analysis of five RCTs suggested acupuncture combined with medicine was more effective than single medicine (Figure 3(c)) on PWL, with a mean difference of 1.26 (95% CI, 0.05 to 2.47; *P* < 0.00001; *I*^2^ = 88%).

#### 3.4.3. Subgroup Analysis according to Animal Species Based on PWL

**Figure 4:**
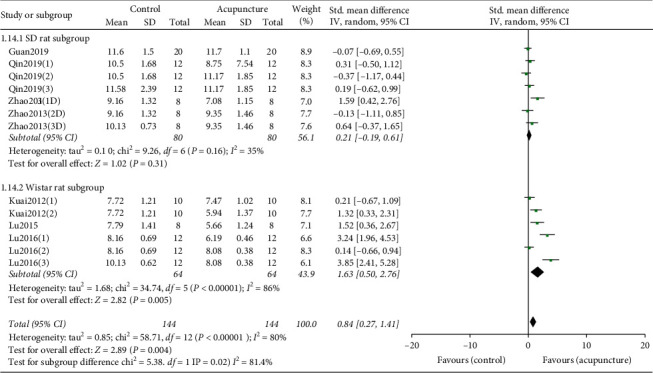
Forest plot of subgroup analysis according to animal species based on PWL. SD rat subgroup: forest plot of SD rat subgroup analyzation comparing PWL between acupuncture and control group. Wistar rat subgroup: forest plot of Wistar rat subgroup analyzation comparing PWL between acupuncture and control group. The black diamond represents the point estimate and 95% confidence interval. The green block at the point estimate of intervention effect with a horizontal line extending either side of the block represents a study. The area of the block indicates the weight assigned to that study in the meta-analysis while the horizontal black line depicts the 95% confidence interval. Statistical method is inverse variance. Random effects model was employed as analysis model. The effect measure was standard mean difference.

To investigate the source of heterogeneity, we developed subgroup analysis according to animal species based on PWL (Figure 4). The heterogeneity decreased after subgroup analysis, which indicates that animal species maybe one of the sources of heterogeneity.

#### 3.4.4. Acupuncture's Effect on Body Weight

**Figure 5:**
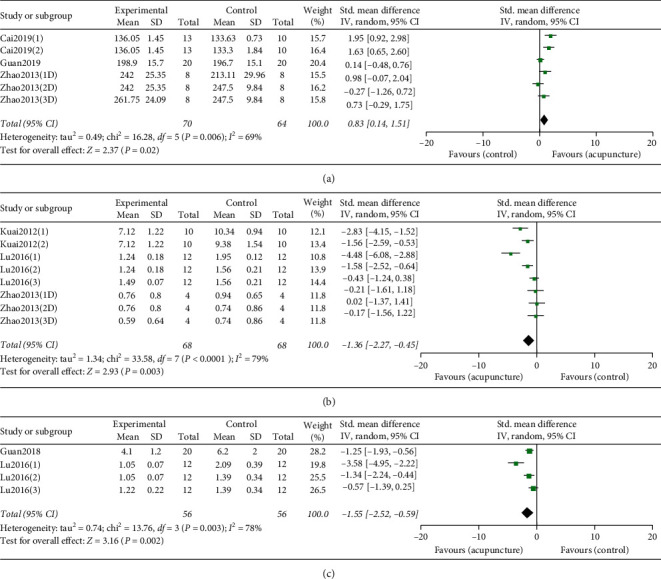
(a). Forest plot comparing body weight between acupuncture and model. (b). Forest plot comparing spinal cord GFAP between acupuncture and medicine. (c). Forest plot comparing spinal cord IL-1*β* between single medicine and medicine combined with acupuncture. The black diamond represents the point estimate and 95% confidence interval. The green block at the point estimate of intervention effect with a horizontal line extending either side of the block represents a study. The area of the block indicates the weight assigned to that study in the meta-analysis while the horizontal black line depicts the 95% confidence interval. Statistical method is inverse variance. Random effects model was employed as analysis model. The effect measure was standard mean difference.

Meta-analysis of six RCTs suggested acupuncture (or acupuncture combined with medicine) was prior to control group (including single medicine, sham/placebo acupuncture, and wait list group) in improving model animals' body weight (Figure 5(a)), with a mean difference of 0.83 (95% CI, 0.14 to 1.51; *P* = 0.006; *I*^2^ = 69%).

#### 3.4.5. Acupuncture's Effect on GFAP

Meta-analysis of eight RCTs showed acupuncture (or acupuncture combined with medicine) could decrease model animals' spinal cord GFAP significantly compared to control group (including single medicine, sham/placebo acupuncture, and wait list group) (Figure 5(b)), with a mean difference of −1.36 (95% CI, −2.27 to −0.45; *P* < 0.00001; *I*^2^ = 79%).

#### 3.4.6. Acupuncture's Effect on IL-1*β*

Meta-analysis of four RCTs suggested acupuncture (or acupuncture combined with medicine) could decrease model animals' spinal cord IL-1*β* significantly compared to control group (including single medicine, sham/placebo acupuncture, and wait list group) (Figure 5(c)), with a mean difference of −1.55 (95% CI, −2.52 to −0.59; *P* = 0.003; *I*^2^ = 78%).

### 3.5. Studies that Cannot Be Pooled by Meta-Analysis

Of the 12 studies included in the qualitative synthesis [Table 5], 3 explored the EA effects on morphine tolerance [35, 36, 42]. These researches showed that compared to bone cancer pain (BCP) group, morphine tolerance group, and sham EA group, EA can improve mechanical pain threshold in rats with bone cancer pain significantly. What is more, the analgesia effect of EA at different time point on morphine tolerance in rats with BCP showed that early intervention of EA stimulation before the formation of morphine tolerance could delay it in rats with BCP [35]; EA stimulation after the formation of morphine tolerance could partially reverse it in rats with BCP. Of the eight studies [21, 23, 24, 26, 30, 35, 36, 42], comparing the effect of EA with sham EA on CIBP rats' thermal pain threshold or mechanical pain threshold, seven studies [21, 23–25, 30, 35, 36] showed that EA is prior to SEA in relieving CIBP. One study [24] showed that neither single nor multiple EA treatment displayed significant analgesia effect on CIBP. Two studies [25, 30] explored how the scheduling regimens and parameters influence the effect of EA on CIBP. One study [25] demonstrated acupuncture at any time showed immediate antinociceptive effect, but acupuncture twice weekly beginning on implantation day 3 or prophylactically three times prior to implantation produced the most robust and longest lasting antinociceptive effect. What is more, gender difference was found in analgesic effect of acupuncture. Another study [30] indicated that EA treatment once had a good analgesic effect on CIBP; the analgesic effect is not related to EA frequency. For EA treatment for a long time, the analgesic effect of EA treatment once every other day is better than EA treatment once a day. Two studies [20, 22] indicated that EA alone has no effect on CIBP, but EA can make small dose of Celebrex, which appears useless in relieving CIBP, exhibiting prominent analgesic effect. One study [38] demonstrated that both EA and morphine were effective on CIBP, while EA's analgesic effect was weaker compared with morphine.

### 3.6. Publication Bias

**Figure 6:**
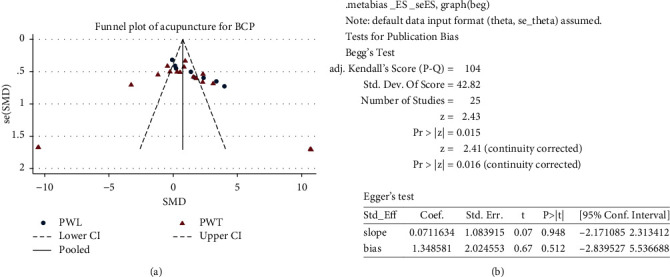
(a). Depiction of publication bias for the paw withdrawal threshold and paw withdrawal latency between acupuncture and control. The red triangle represents the study numbers referring to the paw withdrawal threshold. The blue circle represents the study numbers referring to the paw withdrawal latency. The dashed lines indicated the triangular region with pseudo 95% confidence interval. (b). Begg's and Egger's test for publication bias. CI: confidence interval; SMD: standard mean difference; PWT: paw withdrawal threshold; PWL: paw withdrawal latency.

To address publication bias, we performed funnel plots (Figure 6(a)) for the studies included in quantitative synthesis. No asymmetric pattern was seen. Both Egger (*P* = 0.016) and Begg's test (*P* = 0.512) showed that there was no publication bias (Figure 6(b)).

STRICTA 2010 checklist showed that the following items were reported well: style of acupuncture, reasoning for treatment provided, extent to which treatment was varied, number of needle insertions per subject per session, names of points used, needle stimulation, needle retention time, number of treatment sessions, frequency and duration of treatment sessions, details of other interventions administered to the acupuncture group, and precise description of the control or comparator. At least 22 of the 24 trials reported detailed information of the above items. 11 trials reported the depth of insertion, 8 trials described response sought, and 1 trial demonstrated needle type. None of the included studies prescribed the following items: setting and context of treatment; description of participating acupuncturists; and rationale for the control or comparator in the context of the research question. So future studies should provide more information about the setting and context of treatment, acupuncturists' background, and rationale for the control or comparator in the context of the research question.

As SYRCLE checklist, the following items were reported well: baseline characteristics, selective report, and random housing animals. None of the following items were reported in any of the included studies: allocation concealment, caregivers and/or investigators' blinding, and random outcome assessment. Some items were poorly reported: sequence generation, outcome assessor blinding, incomplete outcome data influence, and other sources of bias.

## 4. Discussion

The object of this systematic review and meta-analysis is to evaluate the effectiveness and safeness of acupuncture in treating cancer-induced bone pain. The meta-analysis found that, compared to model group, EA could increase CIBP animals' PWT and PWL significantly and, compared to medicine treatment, EA has no less effect on PWL. As combined with medicine, EA could enforce single medicine's effect in terms of PWT and PWL. Compared to placebo control group or other treatment, EA increases CIBP animas' body weight. Meanwhile, the mechanical research indicates acupuncture could reduce CIBP animals' spinal cord GFAP and IL-1*β* effectively.

The remaining 12 trials did not pool with meta-analysis due to the fact that no detailed data could be acquired. Three of these researches showed that EA could delay and partially reverse morphine tolerance in rats with BCP. Of the eight studies comparing the effect of EA with sham EA on CIBP rats' PWT and PWL, seven showed that EA is prior to SEA in relieving CIBP. One study showed that neither single nor multiple EA treatment displayed significant analgesia effect on CIBP. One study demonstrated acupuncture at any time showed immediate antinociceptive effect, but the most robust and longest lasting antinociceptive effect is only seen as acupuncture twice weekly beginning on implantation day 3 or prophylactically three times prior to implantation produced. Additionally, sex differences were found. Another study indicated that EA treatment once had a good analgesic effect on CIBP, which was not influenced by EA frequency. As the long time treatment, the analgesic effect of EA once every other day was better than EA treatment once a day. Two studies indicated that rats with CIBP cannot benefit from EA alone, but EA can strengthen small dose Celebrex's analgesic effect. One study demonstrated that both EA and morphine were effective on CIBP, while EA's analgesic effect was weaker compared with morphine.

The major implications for future experimental studies are as follows: firstly, the future animal studies should be designed referring to the SYRCLE to minimize risk of bias and increase the quality of study. Secondly, the future articles should provide the background of acupuncturist, due to the fact that the experience of handlers may influence the efficacy of acupuncture greatly. Thirdly, future studies should increase the sample size to get more reliable conclusion. Lastly, the current evidence of our research indicates that CIBP rats benefit from EA, and maybe clinician can employ EA as a conservative treatment for patients who suffer from caner-induced bone pain.

The key strength of this study is that we have used a meta-analysis drawing on an individual animal data from randomized controlled trials of acupuncture for cancer-induced bone pain, which found that EA was superior to both sham and nonacupuncture controls for cancer-induced bone pain condition. However, there were several limitations in this systematic review. Only 5 of the 24 studies were published in English; the remaining studies were published in Chinese. A great majority of the included studies were carried out by Chinese researchers. These elements may induce publication bias. The treatment durations of each study vary greatly. The shortest treatment period was 5 days, while the longest was 20 days. The modeling methods used in the articles were variable. 19 studies used the Walker256 strains to induce neoplasm, 2 studies used the AT-3.1 to induce neoplasm, 1 study used K7M2 to induce neoplasm, and 2 articles used MRMT-1 to induce neoplasm. The above elements may increase the heterogeneity of the meta-analysis. Only 4 RCTs mentioned outcome assessor blinding, which may induce selective bias. No article described whether the caregivers and/or investigators were blinded from knowledge of which intervention each animal received during the experiment, which may contribute to subjective bias. There was no study reporting the calculation of sample size. Little included studies mentioned the detailed methods of randomization. The above factors greatly reduce the quality of the included studies, which is the key stone of the quality of meta-analysis. So more high quality and larger sample size RCTs are necessary to get a reasonable conclusion.

## 5. Conclusion

As far as we know, this is the first systematic review and meta-analysis to summarize acupuncture for cancer-induced bone pain in animal model. In brief, this meta-analysis and systematic review indicates that, in animal models, electroacupuncture can be used to relieve CIBP, or at least as an assistant treatment. Given the poor quality of included trials, the current existing evidence allows limited conclusions to be reached through comparing acupuncture and medicine, and additional trials are needed to improve the reliability of these findings. Future animal studies should be designed under the guidance of the STRICTA 2010 checklist and SYRCLE risk of bias tool to increase study's quality. Besides, clinical studies are necessary to assess the effect of acupuncture on patients suffering from CIBP.

## Figures and Tables

**Table 1:** Detailed information of animal models included in the study.

Study ID	Objects	Cell strain	Carcinoma cell	Species	Inject location
Qiliang [20]	Rat	Walker256	MGCC	Wistar/F	Tibia
Zhang et al [21]	Rat	AT-3.1	PCC	Copenhagen/M	Tibia
Qiliang et al. [22]	Rat	Walker256	MGCC	Wistar/F	Tibia
Zhang et al. [23]	Rat	AT-3.1	PCC	Copenhagen/M	Tibia
Li [24]	Rat	Walker256	MGCC	Wistar/F	Tibia
Smeester et al. [25]	Mouse	K7M2	Osteosarcoma cell	BALB/c 104 female 166 male	Hind paw
Kuai et al. [26]	Rat	Walker256	MGCC	Wistar/F	Tibia
Si-ma et al. [27]	Rat	Walker256	MGCC	SD/F	Tibia
Zhao et al. [28]	Rat	Walker256	MGCC	SD/F	Tibia
Zhao et al. [29]	Rat	Walker256	MGCC	SD/F	Tibia
Du et al. [30]	Rat	Walker256	MGCC	SD/F	Tibia
Du et al. [31]	Rat	Walker256	MGCC	SD/F	Tibia
Lu et al. [32]	Rat	Walker256	MGCC	Wistar/F	Tibia
Lu et al. [33]	Rat	Walker256	MGCC	Wistar/F	Tibia
Sima et al. [34]	Rat	Walker256	MGCC	SD/F	Tibia
Fu et al. [35]	Rat	Walker256	MGCC	SD/F	Tibia
Fu et al. [36]	Rat	MRMT-1	MGCC	SD/F	Tibia
Guan et al. [37]	Rat	Walker256	MGCC	SD/M	Tibia
Liang et al. [38]	Rat	Walker 256	MGCC	SD/F	Tibia
Liang et al. [39]	Rat	Walker256	MGCC	SD/M	Tibia
Qin et al. [40]	Rat	Walker256	MGCC	SD/M	Tibia
Cai et al. [41]	Rat	Walker256	MGCC	SD/F	Tibia
Shi et al. [42]	Rat	MRMT-1	MGCC	SD/F	Tibia
Guan et al. [43]	Rat	Walker256	MGCC	SD/M	Tibia

MGCC = mammary gland cancer cell; PCC = prostate cancer cell; *M* = male; F = female.

**Table 2:** Characteristics of included studies.

Study ID	Treatment/No.	Control/No.	Acupoints	Stimulator parameters	No. of treatments	Outcomes	Side effects	*P*
Qiliang [20]	EA(12) EA + celecoxib(12)	Model(10) model + celecoxib(12)	ST36, BL60	QID, 4/60HZ, 1 mA, 30 min/time	10	PWT, PWL BW	NR	*P* > 0.05 *P* < 0.05
Zhang et al. [21]	EA(7)	SEA(7)	GB30	QD, 10 Hz/2 mA, 30 min/time	5	PWLIL-1*β*	NR	*P* < 0.05
Zhang et al. [23]	EA(7)	SEA(7)	GB30	QD,10 Hz/2 mA, 30 min/time	5	PWLPWPT	NR	*P* < 0.05
Qiliang et al. [22]	EA(12) EA + celecoxib (12)	Model(12) Celecoxib(12)	ST36, BL60	QD, 4/Hz,1 mA,30 min/time	20	PWT	NR	*P* < 0.05
Li [24]	EA(16) EA	Model(8) Model	ST36/BL60 and ST36/GB30 ST36/GB30	QID,15 Hz, 2-5 mA, 30 min/time, 15 Hz,2-5 mA, 30 min/time,	6	PWT	NR	*P* > 0.05
					10	PWT		*P* > 0.05
Smeester et al. [25]	EA(97)	Model(39) Sham EA(67)	ST36	2/week,4 Hz,30 min/time	6	PWT	NR	*P* < 0.01
Kuai et al. [26]	EA(20)	Model(10) Morphine(10)	L3-L5 Jiaji	QD,2/100 Hz,30 min/time	6	PWL	NR	*P* < 0.01
Zhao et al. [28]	EA(10)	model(10)	BL2	QD,2/100 Hz, 0.3 mA,13 min/time	12	PWT, PWL, BW	NR	*P* < 0.05
Zhao et al. [29]	EA(8) EA(8) EA + Zoledronic acid(8)	Model(8) Zoledronic acid(8) Zoledronic acid(8)	BL2	QD,2/100 Hz, 3 mA, 6.5 min/time	12	BW, PWL PWT	NR	*P* < 0.05
Si-ma et al. [27]	EA(20)	Morphine(10)	ST36, SP6	QD,2/100 Hz,0.5–1.5 mA, 30 min/time	9	PWT	NR	*P* < 0.01
Du et al. [30]	EA(42)	Model(7)	ST36, BL60	1-2 mA,30 min/time	7	PWT	NR	*P* < 0.01
Du et al. [31]	EA(8)	Model (8)	ST36, GenDuan	QID,2 Hz,1-2 mA,30 min/time	7	PWT	NR	*P* < 0.05
Lu et al. [32]	EA(12) EA + morphine(12)	Model(12) Morphine(12)	L3-5JiaJi	QD, 2/100 Hz, 2 mA, 30 min/time	6	PWL, GFAP, IL-1*β*	NR	*P* < 0.01
Lu et al. [33]	EA(8)	Model(8)	L3-5JiaJi	QD, 2/100 Hz, 30 min/time	6	PWL	NR	*P* < 0.05
Sima et al. [34]	EA(8) EA + morphine tolerance(8)	SEA(8) Model(8) Morphine tolerance	ST36, SP6	QD,2/100 Hz, 0.5–1.5 mA, 30 min/time	9	PWT	NR	*P* < 0.01
Fu et al. [35]	EA I (8) EA II (8)	Model(8) Morphine tolerance(8) SEA(11)	ST36, BL60	QD,2/100 Hz, 2/100 Hz, 0.5–1.5 mA, 30 min/time	7/18	PWT	NR	*P* < 0.05
Fu et al. [36]	EA(6)	Model(8) Morphine tolerance(8) SEA(6)	ST36, BL60	QD, 2/100 Hz, 0.5–1.5 mA, 30 min/time	7	PWT	NR	*P* < 0.01
Guan et al. [37]	EA + herbal plaster (20)	Herbal plaster(20)	ST36, BL60	QID, 2/100 Hz, 30 min/time,	6	PWT, IL-1*β*	NR	*P* < 0.05
Liang et al. [39]	EA(15)	Model(13) Morphine(13)	ST36, BL60	QID, 2/100 Hz, 0.5-1.0-1.5 mA,30 min/time	8	PWT	NR	*P* < 0.01
Liang et al. [38]	EA(13)	Model(10) Morphine(10)	ST36, BL60	QID, 2/100 Hz, 0.5–1.5 mA, 30 min/time	8	PWT	NR	*P* < 0.01
Qin et al. [40]	EA(12) EA + Herbal plaster(12)	Model(12) plaster(12)	ST36, BL60	QD, 2/100 Hz,0.5–1.5mA30 min/time	12	PWL, PWT	NR	*P* < 0.01
Cai et al. [41]	EA(13)	Model(10) Morphine(10)	ST-36, BL60	QID,2/100 Hz,0.5–1.5 mA,30 min/time	8	PWT, BW	NR	*P* < 0.05
Shi et al. [42]	EA(11)	Model(8) Morphine tolerance(11) SEA(9)	ST-36, BL60	QD, 2/100 Hz,0.5–1.5 mA, 30 min/time	7	PWT	NR	*P* < 0.05
Guan et al. [43]	EA + herbal medicine (20)	Herbal medicine (20)	ST36, GenDuan	QD,1-2 mA,30 min/time	20	BW, PWL	NR	*P* < 0.05

EA = electroacupuncture; PWT = paw withdrawal threshold; PWL = paw withdrawal latency; BW = body weight; PWPT = paw withdrawal pressure threshold; GFAP = glial fibrillary acidic protein; IL-1*β* = interleukin-1*β*; NR = no report.

**Table 3:** STRICTA 2010 checklist for the included studies.

Study ID	1			2							3		4		5	6	
	*a*	*b*	*c*	*a*	*b*	*c*	*d*	*e*	*f*	*g*	*a*	*b*	*a*	*b*		*a*	*b*
Zhang et al. [21]	Y	Y	Y	Y	Y	Y	Y	Y	Y	U	Y	Y	Y	N	N	N	Y
Qiliang [20]	Y	Y	Y	Y	Y	Y	Y	Y	Y	N	Y	Y	Y	N	N	N	Y
Zhang et al. [23]	Y	Y	Y	Y	Y	Y	Y	Y	Y	U	Y	Y	Y	N	N	N	Y
Qiliang et al. [22]	Y	Y	Y	Y	Y	Y	Y	Y	Y	N	Y	Y	Y	N	N	N	Y
Li [24]	Y	Y	Y	Y	Y	N	Y	Y	Y	N	Y	Y	Y	N	N	N	Y
Smeester et al. [25]	Y	Y	Y	Y	Y	Y	N	Y	Y	U	Y	Y	Y	N	N	N	Y
Kuai et al. [26]	Y	Y	Y	Y	Y	Y	N	Y	Y	U	Y	Y	Y	N	N	N	Y
Zhao et al. [28]	Y	U	Y	Y	Y	Y	N	Y	Y	U	Y	Y	Y	N	N	N	Y
Si-ma et al. [27]	Y	Y	Y	U	U	N	N	Y	Y	N	Y	Y	Y	N	N	N	Y
Zhao et al. [29]	Y	Y	Y	Y	Y	Y	N	Y	Y	N	Y	Y	Y	N	N	N	Y
Du et al. [30]	Y	Y	Y	Y	Y	N	N	Y	Y	N	Y	Y	Y	N	N	N	Y
Du et al. [31]	Y	Y	Y	Y	Y	N	N	Y	Y	N	Y	Y	Y	N	N	N	Y
Lu et al. [32]	Y	Y	Y	Y	Y	Y	N	Y	Y	U	Y	Y	Y	N	N	N	Y
Lu et al. [33]	Y	Y	Y	U	U	N	N	Y	Y	N	Y	Y	Y	N	N	N	Y
Sima et al. [34]	Y	Y	Y	Y	Y	Y	N	Y	Y	U	Y	Y	Y	N	N	N	Y
Fu et al. [35]	Y	Y	Y	Y	Y	N	N	Y	Y	N	Y	Y	Y	N	N	N	Y
Fu et al. [36]	Y	Y	Y	Y	Y	N	N	Y	Y	U	Y	Y	Y	N	N	N	Y
Liang et al. [38]	Y	Y	Y	Y	Y	Y	N	Y	Y	Y	Y	Y	Y	N	N	N	Y
Liang et al. [39]	Y	Y	Y	Y	Y	N	N	Y	Y	U	Y	Y	Y	N	N	N	Y
Guan et al. [37]	Y	Y	Y	Y	Y	N	Y	Y	Y	N	Y	Y	Y	N	N	N	Y
Qin et al. [40]	Y	Y	Y	Y	Y	Y	Y	Y	Y	N	Y	Y	Y	N	N	N	Y
Cai et al. [41]	Y	Y	Y	Y	Y	Y	Y	Y	Y	U	Y	Y	Y	N	N	N	Y
Shi et al. [42]	Y	Y	Y	Y	Y	N	N	Y	Y	U	Y	Y	Y	N	N	N	Y
Guan et al. [43]	Y	Y	Y	Y	Y	N	N	Y	Y	N	Y	Y	Y	N	N	N	Y

Y = yes; N = no; U = unclear.

**Table 4:** SYRCLE risk of bias tool for included studies.

Study ID	1	2	3	4	5	6	7	8	9	10	Total
Zhang et al. [21]	N	Y	N	Y	U	U	Y	U	Y	U	4Y4U2N
Qiliang [20]	U	Y	U	Y	U	N	U	Y	Y	U	4Y5U1N
Zhang et al. [23]	U	Y	U	Y	U	U	Y	N	Y	U	4Y5U1N
Qiliang et al. [22]	U	Y	U	Y	U	N	U	Y	Y	Y	5Y4U1N
Li [24]	U	Y	U	Y	U	N	U	Y	Y	U	4Y5U1N
Smeester et al. [25]	N	Y	N	Y	U	N	Y	N	Y	N	4Y1U5N
Kuai et al. [26]	Y	Y	U	Y	U	N	U	N	Y	N	4Y3U3N
Zhao et al. [28]	Y	Y	U	Y	U	U	U	U	Y	U	4Y6U
Si-ma et al. [27]	Y	Y	U	U	U	U	U	U	Y	U	3Y7U
Zhao et al. [29]	U	Y	U	Y	U	N	U	Y	Y	U	4Y5U1N
Du et al. [30]	U	Y	U	Y	U	N	U	Y	Y	Y	5Y4U1N
Du et al. [31]	U	Y	U	Y	U	N	Y	N	Y	N	4Y4U2N
Lu et al. [32]	Y	Y	U	Y	U	N	U	Y	Y	U	5Y4U1N
Sima et al. [34]	Y	Y	U	U	U	U	U	U	Y	U	3Y7U
Lu et al. [33]	U	Y	U	Y	U	U	U	U	Y	Y	4Y6U
Fu et al. [35]	U	Y	U	Y	U	U	U	U	Y	Y	4Y6U
Fu et al. [36]	U	Y	U	Y	U	N	U	U	Y	U	3Y6U1N
Liang et al. [38]	U	Y	U	Y	U	U	U	U	Y	U	3Y7U
Liang et al. [39]	U	Y	U	Y	U	N	U	U	Y	N	3Y5U2N
Guan et al. [37]	U	Y	U	U	U	N	U	Y	Y	U	3Y6U1N
Qin et al. [40]	N	Y	N	Y	U	U	U	U	Y	U	3Y5U2N
Shi et al. [42]	Y	Y	U	Y	U	N	U	N	Y	N	4Y3U3N
Cai et al. [41]	U	Y	U	Y	U	N	U	N	Y	N	3Y4U3N
Guan et al. [43]	U	Y	U	Y	U	N	U	Y	Y	Y	5Y4U1N

Y = yes; N = no; U = unclear.

**Table 5:** Studies that cannot be pooled by meta-analysis.

Study ID	EA group	Control group	Potential mechanism
Qiliang [20]	PWT⟶	PWT ↑ (EA + Celebrex)	X
Qiliang et al. [22]	PWT⟶	PWT ↑ (EA + Celebrex)	X
Zhang et al. [21]	PWL↑	PWL ⟶ (SEA)	EA suppresses the IL-1*β*
Zhang et al. [23]	PWL↑PWPT↑	PWL ⟶ PWPT ⟶ (SEA)	EA decreases PPD mRNA
Li [24]	PWT⟶	PWT ⟶ (SEA)	X
Smeester et al. [25]	PWT↑	PWT ⟶ (SEA)	EA reduces tumor-associated neutrophils and PGE2
Zhao et al. [28]	PWT↑PWL↑	PWT ⟶ PWL ⟶ (Model)	The contents of rats hypothalamic *β-*endorphin were restrained
Du et al. [30]	PWT↑	PWT ⟶ (SEA, model)	X
Fu et al. [35]	PWT↑	PWT ⟶ (SEA) PWT ⟶ (MT)	X
Fu et al. [36]	PWT↑	PWT ⟶ (SEA) PWT ⟶ (MT)	Improve the MOR positive cells expression in nucleus coeruleus
Liang et al. [38]	PWT↑	PWT ↑ (morphine) (EA < morphine)	EA increased splenic Con A-induced T cell proliferation and plasma IL-2 content and increased the percentages of splenic CD3+ CD4+ and CD3+ CD8+ T cell subsets
Shi et al. [42]	PWT↑	PWT ⟶ (Model) PWT ⟶ (SEA) PWT ⟶ (MT)	Increase MOR expression and promote endocytosis of MOR in locus coeruleus region

PWT = paw withdrawal threshold; PWL = paw withdrawal latency; PWPT = paw withdrawal. Pressure threshold; EA = electroacupuncture; SEA = sham electroacupuncture; PPD = preprodynorphin; MT = morphine tolerance; PGE2 = prostaglandin *E*_2_; Con A = concanavalin. A; MOR = *μ*-opioid receptor; ↑ = increase; ⟶ = no change; x = no report.
